# A comprehensive survey on exploring and analyzing COVID-19 mobile apps: Meta and exploratory analysis

**DOI:** 10.1016/j.heliyon.2024.e35137

**Published:** 2024-07-24

**Authors:** Habib Ullah Khan, Yasir Ali, Muhammad Azeem Akbar, Faheem Khan

**Affiliations:** a Department of Accounting and Information Systems, College of Business and Economics, Qatar University, Doha, Qatar; b Shahzeb Shaheed Government Degree College Razzar, Swabi, Higher Education, KP, Pakistan; c Software Engineering Department, Lappeenranta-Lahti University of Technology, 15210, Lappeenranta, Finland; d Department of Computer Engineering, Gachon University, Seongnam-si, 13120, South Korea

**Keywords:** COVID-19, Coronavirus, SARS-CoV-2, Mobile applications

## Abstract

During the current COVID-19 pandemic, many digital solutions around the world have been proposed to cope with the deadly virus but the role of mobile-based applications is dominant one. In Pakistan, during the current COVID-19 pandemic, an array of mobile health applications (apps) and platforms have been launched to grapple with the impacts of the COVID-19 situation. In this survey, our major focus is to explore and analyze the starring role of mobile apps based on the features and functionalities to tackle the COVID-19 disease, particularly in Pakistan. In this study, over fifty (50) mobile apps have been scrapped from the well-known three different sources i.e. Google Play Store, iOS Play Store, and web source. We developed our own data set after searching through the different play stores. We have designed two criteria such that the first criteria are known as eligibility criteria, while the second one is known as assessment criteria. The features and functions of each mobile app are pinpointed and discussed against the parameters of the assessment criteria. The major parameters of assessment criteria are: (i) Home monitoring; (ii) COVID-19 awareness; (iii) contact tracing; (iv) telemedicine; (v) health education; (vi) COVID-19 surveillance; (vii) self-assessment; (viii) security; and (ix) accessibility. This study conducted exploratory analysis and quantitative meta-data analysis by adopting PRISMA guidelines. This survey article is not only discussing the function and features of each COVID-19-centered app in Pakistan, but it also sheds light on the limitations of every mobile app as well. The results of this survey might be helpful for the mobile developers to review the current app products and enhance the existing mobile platforms targeted towards the COVID-19 pandemic. This is the first attempt of its kind to present a state-of-the-art survey of the COVID-19-centered mobile health apps in Pakistan.

## 1 Introduction

Treatment of infectious diseases using medication can be challenging job [1]. Therefore, some precautionary measures must be adopted to avoid the spread of such contagious diseases particularly the COVID-19. Pakistan played phenomenal in combating and controlling the major wide spread of corona virus. Pakistan achieved 3rd position after Hong Kong and New Zealand around the globe in the fight against COVID-19 according to the report issued by the Economic Normalcy Index [2]. According to this report, Pakistan scored 84.4 on a scale ranging from 0 to 100. The efforts of the National Command and Operation Center (NCOC) are commendable as this ranking is achieved with limited resources by the 7th most populous country in the world. Pakistan administrated the COVID-19 vaccine drive to bring an end to the pandemic but still, it was imperative to adhere to Standard Operating Procedures (SOPs) due to the scarcity of vaccination for the people and demographical factors. To address the rising pandemic of COVID-19, the National Health Services (NHS) plan launched an initiative to combat the COVID-19 pandemic. Similarly, to provide information and address the complaints government launched a call and message services on 1166. Moreover, regular mobile Short Message Service (SMS) have been sent to the public as public service messages and developed awareness about this situation. Similarly, the government of Pakistan launched a COVID-19-designated website (https://covid.gov.pk/) for the public to provide updated position analysis and raise the awareness about the preventive measures against COVID-19. But, among all these efforts and initiatives, the role of digital technologies in handling the COVID-19 situation is important to be highlighted. Digital technologies have been employed for numerous purposes such as the detection, prevention, diagnosis, and surveillance of the COVID-19 pandemic [3]. Digital health technologies can help in providing pandemic strategies and reactions in ways that are hard to attain manually [4,5]. They cover many aspects of COVID-19 such as data analysis, prediction, tracing, responding to public health, population surveillance, clinical care, and rapid case identification [6]. Digital technologies not only contributing towards the economic aspects [7] but Machine learning (ML) and data mining techniques have shown some promising results in the healthcare sector [[8], [9], [10]]. In the list of digital technologies, ML solitary have played dominant role over the period of time [11]. While combining ML with big data can also be more effective for making informed optimal decision-making and predictive analytics [12]. Similarly, Edge computing combines IoT for medical services with clinical monitoring devices [13].

Keeping in view the significance of digital technology like other countries, Pakistan also shifted its paradigm from a manual approach towards the digital health technologies. The digital health innovations are centered on addressing issues like maternal, infant, and child health, less immunization insurance coverage, insufficient access to life-saving drugs, infectious disease outbreaks, and the elevating onus of non-communicable diseases. In digital health technology, the leading role is played by mobile health applications in curbing the transmission and prevention of coronavirus. Mobile health (mHealth) apps have played a significant role in mitigating the COVID-19 response [14]. As the number of smartphone users has reached 137 million in Pakistan [15]. According to another report, this figure is around about 176 million [16]. A huge rise in the number of smartphone users is expected in the next couple of years. Mobile technologies provides matchless opportunities in middle and low-income countries around the world. The role of mobile health apps concerning dealing with highly transmittable diseases is of paramount importance among digital health solutions [17,18]. The concept of using mobile-based health applications for the COVID-19 scenario is not a novice deployment but initially, the smartphone-based apps were employed during the Dengue outbreak back in 2011, where a GPS-enabled mobile phone app for Dengue surveillance program was adopted in Lahore to track the infected persons [19]. Similarly, Sehat Kahani and eDoctor apps were already used for providing remote assistance to patients by healthcare professionals, and the Teeku app was already aimed to provide vaccination and monitoring data to cover vaccination programs well [20]. The vaccination became a tough challenge in the metropolitan city of Karachi during the period of lockdown. To address this challenge, to track and enroll the vaccination status, bio-data of children, and geographical location by using smartphones; which has facilitated the collection of real-time data for the monitoring and evaluation the service delivery [21].

During the COVID-19, mobile apps not only contributed towards the health sector but also made huge impact in the education sector in Pakistan. In Pakistan, pre-service training was imparted to the teachers through the mobile learning technology [22]. The paper provide a platform to change the attitude of students towards digital education in promising ways. These apps provide a lot of information on e-learning resources and materials [23]. Taleemabad app provides game-based learning to the primary level students. But, in this research work our discussion will be limited to highlight those apps that were launched or updated during the COVID-19 pandemic in Pakistan. In Pakistan during the COVID-19 more than 50 mobile health apps were updated for adding more features related to healthcare and 18 apps were merely launched only for COVID-19 specific purposes. In the list of the mobile apps, the most significant and popular one is known as COVID-19 Gov PK app. It includes features like self-home assessment, COVID-19 information status and awareness, notification alerts and complete detail about hospitals. Although, privacy and security are some concerns related with this app. In Pakistan the government as well as private sectors played equal role in introducing various kinds of COVID-19 centered mobile apps. National Information Technology Board (NITB) and Agha Khan University Hospital (AKUH) are the top applications developing bodies. The most re-known app-COVID-19 Gov PK is launched by government through the collaboration of NITB [24]. Another recently app launched by NITB for tracking the records of overseas passengers entering in Pakistan is known as Pass Track app. Similarly, the mobile health apps presented by AKUH are CoronaCheck, Family Hifazat, AKUH Sehat Check and many others. CoronaCheck is the most important COVID-19 app is providing home assessment and information provision in user-friendly fashion for both Android and iOS users [25]. It is providing a good insight and guide about COVID-19 through experts from authentic sources. This app is also aimed to identify the COVID-19 carrier. Other added features are access to WHO and video in Urdu language and online hospital assistance. In Pakistan the role of WhatsApp to deal with COVID-19 pandemic is also of great importance [26]. In Pakistan, the WHO mobile applications also have been given access to all citizens. These apps are WHO Info and OpenWHO: Knowledge for health emergencies. All these apps will be discussed in later section of this article.

To completely understand the overall picture of all mobile apps in Pakistan, an exploratory and cross-sectional analysis of all mobile apps is conducted. Initially our focus was to identify and collect all the mobile health apps that were launched during the COVID-19 outbreak in Pakistan. However, we also considered those apps launched pre-COVID-19 but were updated during the pandemic to meet the COVID-19 requirement. In this review, two criteria have been designed for selection of mobile apps from play stores such as Google play store, Apple app store and Microsoft app store. The first criteria are known as eligibility criteria and second criteria is known as assessment or evaluation criteria. The eligibility criteria are intended to restrict the number of apps and it works as filter in final selection of smartphone apps for study. The second criteria have been applied to study the final selected apps for analysis and discussion. The main evaluation parameters of this criteria are user's rating, no of installations, geographical location, language, app type and size of app. Similarly, the major parameters of assessment criteria are: (i) home monitoring; (ii) COVID-19 awareness; (iii) contact tracing; (iv) telemedicine; (v) health education; (vi) COVID-19 surveillance; (vii) self-assessment; (viii) security; and (ix) accessibility.

A. Contribution

This is a novel approach due to the following major reasons.

• This is the first kind of survey that is focused on highlighting the applications and contributions of mobile phone technology during the COVID-19 pandemic in Pakistan.
• A detailed meta-data analysis and in-depth analysis of the selected corona-related mobile apps in this study has been performed based on two designed criteria i.e. eligibility and assessment criteria. All the mobile apps were checked against these two criteria. All the mobile health apps related to COVID-19 collected in this study are fully explored, analyzed, and discussed based on our evaluation parameters.
• This study has the potential to understand the current research works and future research directions in this area. This study is helpful for the healthcare professionals, mobile subscribers, and mobile app development companies to review their apps for future enhancements.

This paper is composed of the eight remaining sections. Section (II) discusses the related work, section (III) is about the research methodology, section (IV) elaborates on the results of our findings, section (V) is related to the meta-data analysis of mobile apps, the limitations of this research work are discussed in section (VI) is about and (VII) is highlighting the results of this survey, and finally, section (VIII) gives the concluding remarks related to this work.

## 2 Related work

The main focus of this section is to compare the proposed survey with the existing works in the literature. We have made some laborious efforts to highlight all the similar studies that were intended to analyze and explore the role of mobile apps during the COVID-19 pandemic. However, we failed to find any study which have been previously presented to discuss the role of mobile apps during the pandemic in Pakistan. The proposed paper highlight some of the major studies that have been conducted in the different regions of the world.

Islam et al. [27] explored the COVID-19 mobile apps by conducting an exploratory analysis. In this study, the authors included 25 mobile apps by exploring their features and functions. The major element of their evaluation criteria is remote monitoring, patient monitoring, COVID-19 prevention and controlling, awareness, treatment services and mental health support.

Ming et al. [28] analyzed the features and evaluated the content of mobile health apps presented by the different countries during the pandemic. The major features of their study were contact tracing, home monitoring surveillance, COVID-19 information and online consultation.

The study forwarded by Kondylakis et al. [29] uses systematic approach to shed light on the various COVID-19 mobile app features. They evaluated mobile apps according to the parameters like training, information sharing, risk assessment, self-monitoring, contact tracing and home monitoring.

Lee et al. [30] examined the COVID-19 mobile apps that were targeted for East and South-East Asian regions. The authors analyzed the content of selected mobile apps based on COVID-19 testing, public awareness, health monitoring, vaccination, health resources and quarantine monitoring.

Elkhodr et al. [31] performed the content analysis of contact tracing mobile apps. The authors evaluated 13 mobile apps selected from the different countries across the world. The major focus of this study was to check the privacy and usability features of the selected apps in their assessment.

The study conducted by Alanzi et al. [32] reviewed twelve (12) mobile applications selected from the different countries including US, UK, Arabia, Italy, Singapore and India. The major focus of this study is to assess the mobile health applications in light of the criteria. The major element of the criteria features includes Contact tracing, online consultation, COVID-19 information, self-assessment and test reporting.

Montano et al. [33] work is focused on analyzing twenty-four (24) mobile apps in Spain. This work ranks the mobile apps according to analyzing the mobile apps functionalities, features and limitations. This study analyses the COVID-19 mobile apps according to criteria consisting of COVID-19 information, self-evaluation, monitoring, communication with healthcare professionals and case tracking.

Sujarwoto et al. [34] included seven (07) mobile apps according to their inclusion criteria in Indonesia. Authors used systematic approach for analysis of the selected mobile apps for the COVID-19 related features such as COVID-19 information, online consultation and communication, telemedicine and self-risk assessment.

Zhou et al. [35] concudcted their survey based on collecting some important data about the COVID-19 mobile health apps. The major focus of this study was to understand about the user's privacy, role of community and government, situational specificity, possible improvement and strengths of the mobile health technology in China.

Singh et al. [36] work is aimed to forward scoping review of the selected COVID-19 mobile apps from the different countries. The major theme of their study was to explore the extracted mobile apps based on COVID-19 specific functions and features such contact tracing, symptoms monitoring, COVID-19 information and quarantine.

Bass et al. [37] studied the COVID-19 selected mobile apps based on three major criteria elements. The major focus of this study was to highlight the mobile health app development based on corona related features such as contact tracing, self-testing and quarantine monitoring.

The review work performed by Schmeelk et al. [38] is targeted to evaluate the symptoms monitoring functionality of the selected mobile apps. This study used a scale for the evaluation of quality of mobile apps.

The complete summary of the related works in comparison to the proposed work is given in Table 1.

**Table 1:** Comparison of proposed work with the existing studies.

Study	COVID-19 specific features	Methodology	Country/Region	Year
Islam et al. [27]	Remote monitoring, patient monitoring, COVID-19 prevention and controlling, awareness, treatment services and mental health support.	Review	Globally	2020
Ming et al. [28]	Contact tracing, home monitoring surveillance, COVID-19 information and online consultation.	Review	Globally	2020
Kondylakis et al. [29]	Training, information sharing, risk assessment, self-monitoring, contact tracing and home monitoring.	Systematic literature review (SLR)	Globally	2020
Lee et al. [30]	COVID-19 testing, public awareness, health monitoring, vaccination, health resources and quarantine monitoring.	Review	East and South-East Asian regions	2021
Elkhodr et al. [31]	Privacy by design, Privacy implementation and Usability and Users perceptions	Review	Globally	2021
Alanzi et al. [32]	Contact tracing, online consultation, COVID-19 information, self-assessment, test reporting	Review	USA, Italy, Singapore, United Kingdom, Arabia, and India	2021
Montano et al. [33]	COVID-19 information, self-evaluation, monitoring, communication with healthcare professionals, case tracking	Review	Spain	2022
Sujarwoto et al. [34]	COVID-19 information, online consultation and communication, telemedicine and self-risk assessment.	SLR	Indonesia	2022
Zhou et al. [35]	Access to the COVID-19 information, contact tracing, crowd monitoring and receiving health alerts/notifications	Survey	China	2021
Singh et al. [36]	COVD-19 information, contact tracing, home isolation and symptoms monitoring	Review	Worldwide	2022
Bass et al. [37]	Contact tracing, self-testing and quarantine monitoring	Review	India	2020
Proposed work	Home monitoring, Medical education, self-assessment, COVID-19 awareness (information), contact tracing, medical education and telemedicine	Survey	Pakistan	2024

## 3 Research methodology

This section briefly explains the procedure adopted for the collection of mobile apps and the criteria applied for the mobile app selection. The procedure of collecting mobile apps related to COVID-19 is done in a well-organized and systematic way. This research approach follows Preferred Reporting Items for Systematic Reviews and Meta-Analysis) PRISMA guidelines for selecting mobile apps from different sources. The details of the steps i.e., mobile app collection and criteria designing/application are given below.

A. Scrapping mobile apps Play stores

This is the first and most important step of our research methodology. A systematic technique is followed to scrap the mobile apps from the most popular play stores such as Google Play, iOS, and Microsoft app stores. Many sets of keywords were defined to extract all the mobile applications intended for COVID-19 tasks during the outbreak. The list of keywords defined for searching mobile apps is: “Corona apps”, “COVID-19”, “SARS-CoV-2“,” COVID-19 and Coronavirus apps”, “COVID-19 and Corona Self-assessment “, “COVID-19 home monitoring”, “COVID-19 telemedicine app”, “COVID-19 awareness apps” etc.

**Fig. 1:**
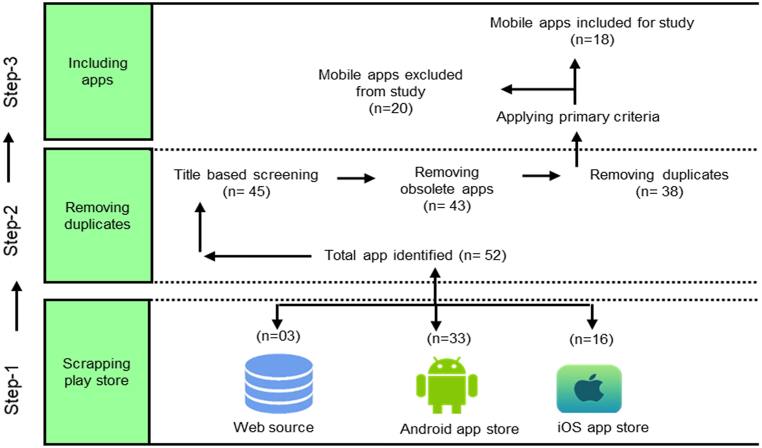
Procedure of extracting mobile apps from play stores.

All the retrieved apps were maintained in a database file that describes the app name, release date, update date, developer, no of installations, user's rating, app size, version number, permission level, and category of app. According to our statistical reports, more than 52 mobile apps were identified. The mobile apps are collected from the play Store such as thirty-three (33) mobile apps are collected from the Google Play Store, 16 mobile apps from iOS, and 03 were collected from web sources. Some of the mobile apps were found available on both platforms such as Android and iOS play store, but the duplicate was removed. After removing the repeated apps, the number of apps was reduced to 43. So, finally, a total of 43 mobile apps were collected from all three mobile app stores as given in Table 2. The complete graphical procedure of identifying, collecting, filtering, and finalizing mobile apps is given in Fig. 1.

**Table 2:** Complete list of mobile apps collected.

No	App name	No of installs	Rating	Size (MB)	Update date	Release date	Permissions	Category
1	COVID-19 Gov PK	500,000+	4.2	6.9	December 1, 2020	March 27, 2020	Location, Storage, Camera, Network	Health and Fitness
2	COVID-19 Care for Media	10,000+	4.3	6.2	March 30, 2021	March 24, 2020	SMS, Network	Communication
3	Pakistan's National Action Plan for COVID-19	50,000+	3.8	12	March 25, 2020	February 18, 2020	No	Health and Fitness
4	Health Assessment PDMA	10,000+	3.6	14	June 16, 2020	March 24, 2020	Location, Internet	Health Education
5	Health Monitoring PDMA	5,000+	3.5	5.0	April 14, 2020	March 31, 2020	Location, Network	Productivity
6	Pakistan Red Crescent Sindh	500+	3	37	April 29, 2021	Apr 4, 2020	Location, Storage, Media, Microphone, Internet	Communication
7	OpenWHO: Knowledge for Health Emergencies	1,000,000+	4.3	18	November 23, 2020	May 17, 2017	Media, Storage, Wi-Fi, Internet, Files	Health Education
8	WHO Info	500,000+	4	12	April 29, 2021	Apr 13, 2020	Storage, Calendar, Media, Network	News and Magazines
9	CoronaCheck	100,000+	3.6	4.5	April 4, 2020	March 30, 2020	Full network access	Medical
10	Family Hifazat	10,000+	4.3	52.3	January 12, 2021	Apr 18, 2018	Device ID, Call info, Phone, Media Microphone, Storage, Location, Wi-Fi, Camera, Internet	Health and fitness
11	AKUH Sehat Check	10,000+	4.1	10	December 2, 2020	May 03, 2020	Phone, Device ID, Wi-Fi, Network Internet	Health and fitness
12	AKUH Patient Care	100,000+	2.6	18	June 15, 2020	Oct 27, 2016	Contact, Location, Camera, Storage, Media, Internet	Medical
13	AKUH, K-JCIA	5,000+	4.0	2.6	August 15, 2018	July 27, 2018	Internet	Health Education
14	PurUmeed Aaghaz	100+	3+	2.7	November 20, 2019	November 19, 2019	Internet	Health & Fitness
15	MyPatients@aku	10,000+	4.5	80	April 20, 2021	Sep 15, 2016	Phone, Device ID, Wi-Fi, Network Internet, Contact, Location, Camera, Storage Media	Medical
16	FMIC MyPatients@aku	100+	3+	2.3	April 17, 2019	Apr 16, 2019	Full network access	Medical
17	EthAKUL	100+	3+	4.5	November 1, 2018	May 14, 2018	Storage, Media, Camera, Network	Medical
18	AKUH Update Patient Location	1000+	3+	4.15	Nov 18, 2016	Nov 2, 2016	Camera, Network	Medical
19	Shoukat Khanum App	10,000+	3+	14.4	April 15, 2021	Oct 25, 2017	Camera, Storage, Location, Network	Medical
20	Track Pass	500,000+	3.6	11.34	May 7, 2021	Jul 10, 2020	Camera, Storage, Full network access	Health & Fitness
21	CL PCR Report Scanner	5,000+	3.7	13	December 22, 2020	Nov 23, 2020	Media, Storage, Camera, Network	Health & Fitness
22	ChughtaiLab	100,000+	4.1	36	May 20, 2021	Sep 30, 2014	Camera, Storage, Location, Network	Health & Fitness
23	HealthSolutions	10,000+	4.3	92	March 2, 2021	Feb 8, 2018	Camera, Storage, Location, Network, Calendar, Microphone	Health & Fitness
24	Sehat Kahani corporate	10,000+	4.2	38	March 30, 2021	April 11, 2019	Camera, Storage, Location, Network, Calendar, Microphone, Other	Medical
25	Sehat Kahani App	10,000+	3.8	36	May 21, 2021	April 21, 2019	Camera, Storage, Location, Network, Calendar, Microphone, Other	Medical
26	Marham connect	10,000+	4.3	18.28	May 06, 2021	Jan 24, 2017	Camera, Storage, Location, Network, Calendar, Microphone, Other	Medical
27	Marham Kiosk	100+	3+	3.67	Aug 03, 2017	July 15, 2017	Contacts, Other	Medical
28	Medicall	10,000+	4.3	9.67	April 12, 2021	Mar 18, 2018	Storage, Other	Medical
29	MedIQ: Smart Healthcare	10,000+	4.7	51	May 6, 2021	Apr 29, 2020	Camera, Storage, Location, Network, Calendar, Microphone, Other	Health & Fitness
30	MyPractice by oladoc - Grow your practice	5000+	4.5	20	May 6, 2021	Feb 27, 2018	Camera, Microphone, Storage, Other	Health & Fitness
31	Find My Doctor	1000	3	6.44	Feb 5, 2020	Jan 07, 2016	Camera, Storage, Location, Network, Calendar, Microphone, Other	Health and fitness
32	Pharmapedia Pakstian	100000+	4.2	6.7	Feb 27, 2021	September 11, 2015	Network	Medical
33	Instacare-Pro	1000+	4.9	19	May 31, 2021	Nov 5, 2018	Camera, Microphone, Storage, Other	Health and fitness
34	Dawai-Online medicine and Healthcare	50000+	4.1	55.53	May 31, 2021	Dec 30, 2017	Camera, Storage, Location, Network, Calendar, Microphone, Other	Medical
35	Tele Polyclinic	100+	4.1	9.1	Dec 29, 2020	July 26, 2020	Camera, Storage, Location, Network, Calendar, Microphone, Other	Health and Fitness
36	Healthwire-For doctors	1000+	4.2	15.2	Jan 15, 2021	Oct 14, 2019	Camera, Storage, Location, Network, Calendar, Microphone, Other	Health and Fitness
37	Healthwire	10000+	4.2	17.7	Jun 7, 2021	Apr 24, 2019	Camera, Storage, Location, Network, Calendar, Microphone, Other	Health and Fitness
38	Shifaam Health app	1000+	4.0	24	Jun 16, 2021	Aug 18, 2019	Camera, Storage, Location, Network, Calendar, Microphone, Other	Medical
39	Pak Neghayban	1000+	4.2	3.8	November 28, 2020	June 4, 2020	Location, Other	Medical
40	COVID CHECK PAKISTAN	100	3.3	2.5	June 8, 2020	May 1, 2020	No	Medical
41	Elaj Asan app	1000+	3.4	13.67	Jul 15, 2021	Sep 21, 2020	Camera, Storage, Location, Network, Calendar, Microphone, Other	Medical
42	Elaaj Plus	500+	4.5	6.37	May 27, 2020	April 15, 2020	Camera, Microphone, Storage, Other	Medical
43	Elaj Plus Doctor App	100+	4.8	6.27	10 June 2020	April 12, 2020	Camera, Storage, Location, Network, Calendar, Microphone, Other	Medical

**Fig. 2:**
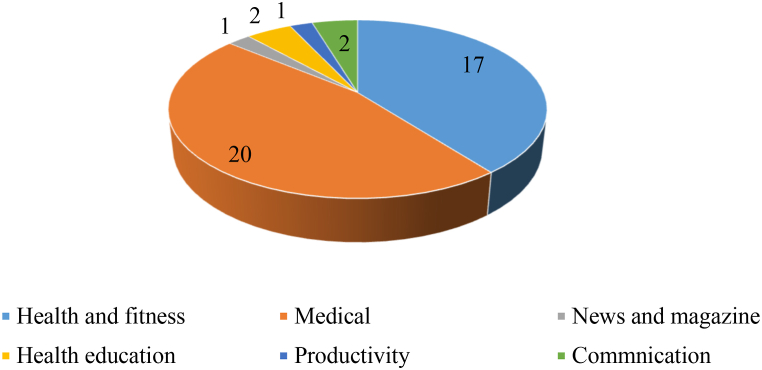
Mobile apps categories.

After collecting 43 mobile health apps from three play stores then exploratory and in-depth analysis has been performed to understand the trend of mobile app development in Pakistan during the COVID-19 outbreak. The categorical division of 43 mobile health apps extracted in this research is given in Fig. 2.

**Fig. 3:**
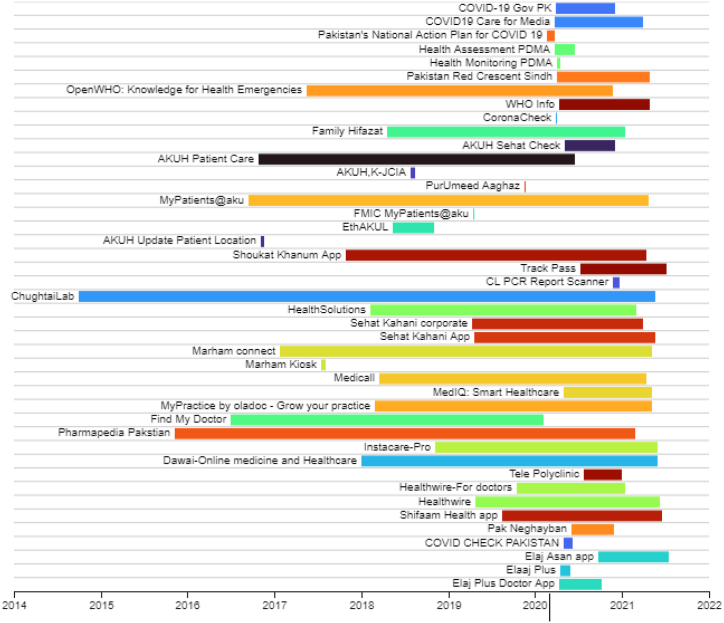
Timeline of mobile apps released and updated during COVID-19 pandemic in Pakistan.

All the mobile apps related to healthcare were identified from the respective play stores. Mobile apps (43) were collected in this research work are given in the Gantt chart diagram in Fig. 3. It shows the release and update dates of mobile apps in their respective play stores. It is important to know about the mobile apps that were released before the pandemic, but COVID-19-specific features were added to the existing apps by updating. It can be seen from this chart that the majority of mobile apps have been released right after the emergence of the COVID-19 pandemic.

B. Criteria designing and application

**Fig. 4:**
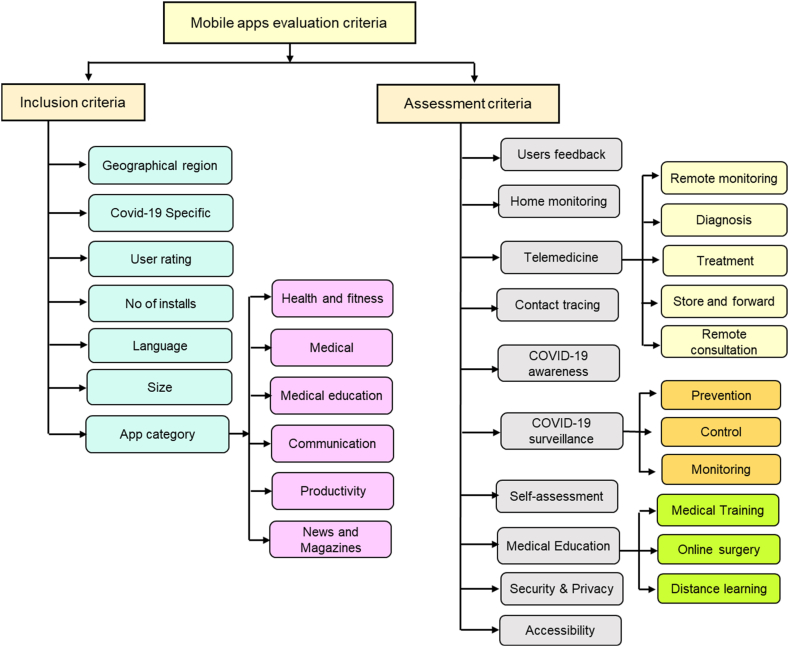
Structure of proposed evaluation criteria.

This is the most important phase of this research work. Designing criteria to discuss different kinds of mobile applications targeted towards COVID-19 is the most valuable and key part of our research study. According to Stoyanov et al. [39], there are five main categories of criteria for the quality assessment of mobile apps according to the Mobile App Rating Scale (MARS) scale. This category of parameters is not related to COVID-19. However, our focus is to build criteria that only focusing and covering on the COVID-19 aspects of mobile health apps. In this regard, we built two criteria such as primary also known as eligibility criteria or inclusion criteria; and secondary criteria also known as assessment or evaluation criteria. The major parameters of inclusion criteria are the size of the app, release date, no of installations, user's rating, geographical region, language, and category of apps. The complete structure of building both criteria is given in Fig. 4.

The category of apps is expressing all the mobile application that belongs to medical education, productivity, medicine, health and fitness, communication news, and magazine. These criteria are applied to the smartphone apps after collecting from the play stores and literature. It works as a filter to reduce the number of mobile applications for final discussion. These criteria are termed eligibility or inclusion criteria. It is applied on all 43 mobile health applications collected from the Play Store. After the application of eligibility criteria, the number of mobile health apps is limited to 18. The complete detail of applying eligibility criteria on mobile apps is given in Table 3.

**Table 3:**
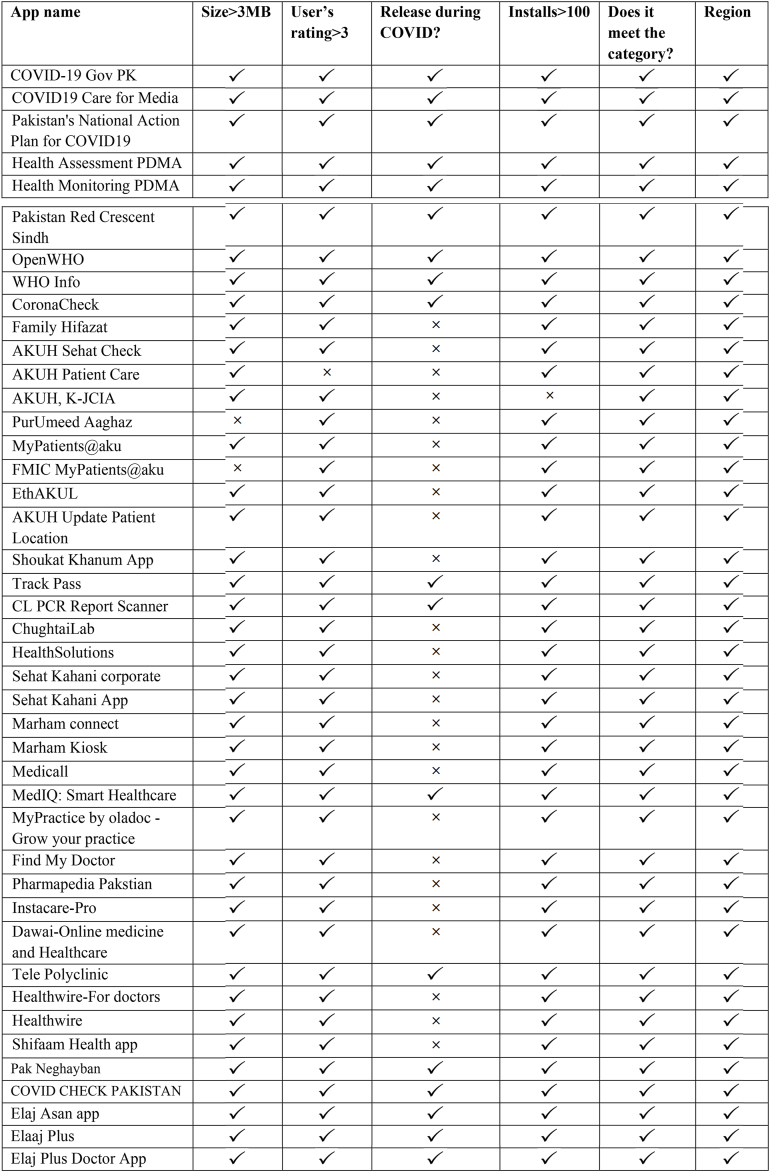
Results of eligibility criteria.

Evaluation or assessment criteria that are applied to discuss the finally selected 18 mobile health applications against the features of criteria. The focus of this criteria is to discuss only the COVID-19-relevant features of each app. The salient mobile features of COVID-19 are highlighted in light of secondary criteria. The major elements involved in the secondary criteria are-self-assessment, contact tracing, COVID-19 awareness, telemedicine, medical training and education, and COVID-19 surveillance. Telemedicine can be described by remote monitoring, diagnoses, treatment, store and forward, and consultation. Similarly, medical education is also broad in scope. It includes medical training, online surgery, and distance learning.

## 4 Results

A list of finally selected eighteen (18) mobile apps are evaluated and discussed based on the features and functions relevant to COVID-19. In this section, the selected mobile apps are discussed based on the features, we selected in the evaluation criteria. The list of mobile apps along with the URLs are given in Table 4.

**Table 4:** List of COVID-19 mobile apps selected for study.

S.N	App name	App URLs
A_1_	Health Monitoring PDMA	https://play.google.com/store/apps/details?id=com.sapphire.HealthAssessmentPDMA
A_2_	Pakistan's National Action Plan for COVID	https://play.google.com/store/apps/details?id=com.nap_pakistan.app
A_3_	Health Assessment PDMA	https://play.google.com/store/apps/details?id=com.sapphire.HealthAssessmentPDMA
A_4_	CoronaCheck	https://play.google.com/store/apps/details?id=com.edu.aku.akuhccheck
A_5_	CL PCR Report Scanner	https://play.google.com/store/apps/details?id=cll.report.scanner.mobile
A_6_	Pass Track	https://play.google.com/store/apps/details?id=com.passtrack.nitb.gov.pk
A_7_	AKUH Sehat Check	https://play.google.com/store/apps/details?id=sehat.check
A_8_	COVID-19 Gov PK	https://play.google.com/store/apps/details?id=com.govpk.covid19
A9	Tele Polyclinic	https://play.google.com/store/apps/details?id=com.innovpk.tpc
A_10_	Pakistan Red Crescent Sindh	https://play.google.com/store/apps/details?id=prcs.dms
A_11_	MedIQ: Smart Healthcare	https://play.google.com/store/apps/details?id=com.homemedics.app
A_12_	COVID19 Care for Media	https://play.google.com/store/apps/details?id=covid19care.virus.coronavirus.corona.sick.marcom.health.pakistan
A_13_	Pak Neghayban	https://play.google.com/store/apps/details?id=com.govpk.paknigehbaan
A_14_	COVID CHECK PAKISTAN	http://ncra.org.pk/covid/
A_15_	WHO Info	https://play.google.com/store/apps/details?id=org.who.infoapp
A_16_	Elaj Asan app	https://play.google.com/store/apps/details?id=com.akhsp.virtualdoc.patient
A_17_	Elaaj Plus	https://play.google.com/store/apps/details?id=com.hatinco.elajplus
A_18_	Elaj Plus Doctor App	https://play.google.com/store/apps/details?id=com.hatinco.elaajplus.doctor

A_1_, A_2_, A_3_ ….A_n_ indicate mobile applications.

The number of installations, rating, app size, level of permission, platform, and language of selected eighteen mobile apps are given in Table 5.

**Table 5:** List of selected apps for study.

App	No of installs	Rating	Size (MB)	Permission level	Platform	Language
A_1_	5,000+	3.5	5	Location	Android	English
				Network		
A_2_	50,000+	3.8	12	No	Android	English
A_3_	10,000+	3.6	14	Location	Android	English
				Internet		
A_4_	100,000+	3.6	4.5	Full network access	Android/iOS	English
A_5_	5,000+	3.7	13	Media	Android/iOS	English
				Storage		
				Camera		
				Network		
A_6_	500,000+	3.6	11.3	Camera	Android/iOS	English/Urdu
				Storage		
				Network access		
A_7_	10,000+	4.1	10	Phone	Android/iOS	English/Arabic
				Device ID		
				Wi-Fi		
				Network		
				Internet		
				Storage		
				Camera		
				Network Access		
A_8_	500,000+	4.2	6.9	Location	Android iOS Web	English Urdu
				Storage		
				Camera		
				Network		
A_9_	100+	4.1	9.1	Camera	Android/Web	English
				Location		
				Microphone		
				Storage		
				Network		
				Other		
A_10_	500+	3	37	Location	Android	English
				Storage		
				Media		
				Microphone		
				Internet		
				Location		
A_11_	10,000+	4.7	51	Location	Android/iOS	English
				Contacts		
				Microphone		
				Storage		
				Phone		
				Network		
				Other		
A_12_	10,000+	4.3	6.2	SMS	Android	English
				Network		
A_13_	1000+	4.2	3.8	Location	Android	English
				Other		
A_14_	100+	3.3	2.5	Storage	Android	English Urdu
				Network		
A_15_	500,000+	4	12	Storage	Android iOS Web	English Arabic French Russian Chinese Spanish
				Calendar		
				Network		
A_16_	1000+	3.4	13.6	Camera	Android	English
				Location		
				Microphone		
				Storage		
				Network		
				Other		
A_17_	500+	4.5	6.3	Camera	Android	English
				Microphone		
				Storage		
				Other		
A_18_	100+	4.8	6.2	Camera	Android	English
				Location		
				Microphone		
				Storage		
				Network		
				Other		

All the features and functions are discussed comprehensively to understand the objective of every mobile app. The complete mapping of mobile health apps for features and functions is given as.

### 4.1 Self-assessment mobile apps

In healthcare sector, the timely diagnosis helps in providing better treatment according to the patients symptoms [40]. This is the reason that the majority of the mobile health apps enabled the people to check their health status. If there are mild symptoms then they can be managed at home, however; if symptoms are severe then they need serious medical attention to visit the hospital. This is an important feature in the context that during COVID-19, the entire healthcare systems were overloaded and it was essential to allow patients with mild symptoms to get treatment at home by connecting with a healthcare physician or expert.

Self-assessment allows to assess the symptoms via smartphone application by using mobile phone and then take decision regarding testing or some other medical care needs. In Pakistan, a specialized app has been introduced by the Agha Khan University for this self-assessment known as CoronaCheck app [41]. It is modified form of Alberta Health Services digital platform; and is available on both Google play store and iOS play store. It has chat board driven by Artificial intelligence (AI) tools to provide user-friendly self-assessment at home. This app uses an assessment tool to ask a series of questions from the users such as travelling history, symptoms and contacting or visiting detail about COVID-infected person. During the COVID-19 situation falsified information and misconceptions were shared on social media and it was very important to negate this information to avoid the chaotic among the people. This app also addresses the same issue by adding educational video in national language (Urdu) to tackle the unverified information on the social platforms. This mobile application provides guidance and precautions related to social distancing suggested by the WHO and national health administration authorities [42]. Similarly, another app known as AKUH Sehat Check launched by AKUH to screen the patients visiting the Agha Khan hospital. This app assesses the patients and healthcare staff health conditions by asking a set of questions from their patients. This app is available on both Apple and Google play store platforms free of cost [43]. COVID-19 Gov PK app is also providing the functionality of self-assessment [44]. This app is available on three digital platforms such as Google play store, Apple app store and in web based form. For self-screening the researchers team of National University of Science & Technology's (NUST) also introduced app known COVID CHECK PAKISTAN. This mobile app is available in two languages such as Urdu and English. This is first app in national language of its kind. COVID CHECK PAKISTAN, is tested for more than over 8,000 screenings in nine different countries such as United State, United Kingdom, United Arab Emirates and Saudi Arabia, within its first two days of its beta rollout. This app allows the citizens to perform self-assess themselves at home by avoiding risks without visiting hospitals [45]. Health Assessment PDMA is also providing the feature of self-assessment and this app is available in three different languages.

During the COVID-19, Pakistan also made an attempt to perform self-assessment and raise awareness by using social media platforms such as WhatsApp and Facebook [46]. Since, the start of pandemic, the polio workers in Pakistan leveraging the messaging apps especially WhatsApp to apprise the people and community about COVID-19. The frontline workers of polio produced videos, digital pamphlets and posters related to COVID-19 for sharing on social media platforms. These messaging apps were not only used by polio workers by also adopted by religious community as well as clerics to aware people about the jeopardy of COVID-19. In Punjab province over 110,000 people were reached out through digital leaflets and posters. In Sindh province, over 200,000 people at risk were helped in this way [47].

In a nutshell, the most prominent mobile apps for self-assessment in Pakistan employed during the coronavirus pandemic are CoronaCheck, AKUH Sehat Check, COVID-19 Gov PK, COVID CHECK PAKISTAN and social media messaging apps.

### 4.2 COVID-19 surveillance mobile apps

COVID-19 surveillance is concept of monitoring the spread of disease to identify the pattern of progression in order to apply control and prevent measures [48]. It is considered as the most vital constituent of public medical care practice [49]. For this purpose, the COVID-19 surveillance apps are targeted to monitor the spread of coronavirus and to detect the progress of probable suspected and confirmed cases based on the data extracted from different data sources. Majority of government from various countries have accelerated to deploy smartphone based surveillance programs in response to the COVID-19 pandemic. The major focus of mobile based surveillance technologies is to track movement of people, contacting, exposure to disease, enforcing quarantine, track symptoms and validate the health status of citizens in order to promote health and provide response to current COVID-19 pandemic and future strategies for next epidemic [50]. The concept of adopting the mobile based surveillance program is a not a new deployment but the mobile technologies based surveillance program in rural communities in Pakistan was initially launched during the hepatitis virus back in 2014 [51]. Pakistan also adopted digital spy surveillance technology to identify the hotspot and densely infected areas [52]. In Pakistan during pandemic, many smartphone apps have been launched to keep a vigilant eye on the current pandemic. Among these apps, COVID-19 Gov PK plays a leading role to monitor and track Coronavirus. In this regard, the NHS have launched a web based platform that is available at (https://covid.gov.pk/) to monitor and track the coronavirus infected patients. Health Assessment PDMA App is helpful for healthcare officials and public to provide real time information related to those emerging area that are at risk due to COVID-19. It assists in better management, prevention and treatment purposes. This app is available in three different languages- Urdu, English and Sindhi [53].

To reach out to the maximum infected people, Pakistan activated the surveillance units at Federal and district level. Similarly, for strict surveillance, Government of Pakistan also initiated monitoring and contact tracing mechanisms. The team of emergency operation center are following up the incoming passengers to Pakistan. For assessing and tracking the incoming infected COVID-19 passenger to Pakistan, a mobile app was launched by NITB, government of Pakistan. This app is known as Pass Track. It is available in both Urdu and English languages on both digital platforms-google play and iOS play stores. It is mandatory for all incoming travelers to Pakistan and all passengers have to install it by providing all the required information easily and securely through this mobile application. The major purpose of this mobile application is to collect data in digital database to provide centralized tracking and monitoring [54].

During the peak hours, Pakistan suffered from the shortage of bed and ventilators and majority of the hospitals were full with overloaded patients. It was so important for the family members to check the availability of ventilators and beds prior to hospitalizing the critical patients. To address this issue NCOC introduced an app known as Pak Neghayban to keep the track of available ventilators and beds across the hospitals in Pakistan. Initially, l100 hospital were associated with this mobile app. This app is really helpful in emergency situations for healthcare professional, emergency responders and public citizens. Initially, Pak Negihban was added as additional feature in COVID-19 Gov PK app [55] but later on this feature was introduced in a segregated mobile app solely intended for this specific purpose known as Pak Negihban app.

### 4.3 Home monitoring apps

Home monitoring is important for people who are suffering from the chronic disease. In countries like Egypt, Pakistan and China, the number of people suffering from the chronic diseases is very high [56]. This is the reason that during the peak hours of the pandemic, the aged people suffering the chronical diseases such diabetes, hypertensions, asthma, depression, mental health issues and cardiovascular diseases were required a special medical attention 24/7 and isolation at home. Hospital were also overloaded due to heavy influx of the infected people. At this stage, people suffering from mild symptoms of COVID-19 were required to be diagnosed and treated at home. To provide solution to these healthcare challenges, smartphone has been used as vital equipment to provide data to the healthcare professionals in easy and fastest way through the different mobile apps. In this context, the role of smartphone has been phenomenal due to supporting many mobile health apps and multi-features support. During the current pandemic, smartphone has been witness for providing many medical applications [57]. According to study conducted Ming et al. [58], 37.5 % of world mobile health apps were focused on surveillance and home monitoring in this pandemic. According to literature study, in Pakistan 35 % of apps are launched to provide home monitoring services to the patients. The list of mobile apps providing home monitoring feature are CoronaCheck, AKUH Sehat Check, COVID-19 Gov PK, Tele Polyclinic, MedIQ: Smart Healthcare and COVID CHECK PAKISTAN. These mobile apps have been used to bring medical care services to the COVID-19 infected patients at the early stages of infection at home without visiting the hospitals. Among these apps, MedIQ: Smart Healthcare supports more features/services related to home monitoring such as booking of medical practitioner through call. It also offers home imaging services such as X-ray, ECG, hemodialysis and dopplers etc. at home. Online ordering medicines, purchasing medical equipment on rental basis, medicine categorization and virtual hospital services are also the other salient features included in this mobile health application [59].

### 4.4 COVID-19 awareness apps

The knowledge about COVID-19 is limited which has led to the global healthcare challenges [60]. In Pakistan, majority of mobile applications were designed to provide COVID-19 awareness to the public. In Pakistan during the coronavirus pandemic 65 % of COVID-19 mobile apps are providing information about the number of infected, suspected, recovered, hospitalized and death. Among the awareness apps, the most prominent app is COVID-19 Gov PK. This app is providing a complete catalogue of COVID-19 information from district level to the whole state during the last 24 h. The web version of this app is also available for desktop users. Corona Check is another mobile app to raise awareness about the measures to prevent the spread of the coronavirus. This mobile health app played an anchor role during the pandemic, whenever, fabricated and falsified information were shared nationwide to fuel the panic among the citizens. This mobile app negated the misconception stories and combated the unverified information circulating on social platforms [61]. Similarly, Health assessment PDMA app is also providing COVID-19 awareness feature but it is only limited to the Sindh province of Pakistan. For providing COVID-19 specific information Corona tracker is also important mobile app. It also provides complete statistical detail related to COVID-19 of every province based on the data extracted from WHO source. It displays the most up to date data about the current situation and last 7 days' average detail by comparing it with previous week's record. Other apps featuring COVID-19 awareness are Health Monitoring PDMA, COVID19 Care for Media, Pak Neghayban, OpenWHO and WHO Info.

### 4.5 Telemedicine apps

Telemedicine is an emerging concept of delivering the healthcare services through the application of digital technology [62]. With the advent of telemedicine the healthcare professionals became able to provide remote healthcare services [63]. In March 2020, whenever a complete lockdown was imposed in the country then it became very hard for patients to visit hospital due to highly overloaded cases in hospital and scarcity of healthcare professionals. Similarly, just like other countries patients were also intimidated to visit hospital due COVID-19 exposure; during this time telemedicine played central role to assist the patients at the time of need. During the COVID-19 crisis, telemedicine applications were used as alternative to provide healthcare delivery services [64]. This is the reason that at the end of February 2020 all doctors in Pakistan switched to online consultation by affiliating themselves with firms. Federal and provincial governments have opted for telemedicine independently or in collaboration with local health services.

In the main city of Pakistan- Faisal Abad telemedicine service was implemented during the COVID-19 and it produced some promising results [65]. In Pakistan the telemedicine services are broken into three different categories: (1) Dashboards and real time registries (2) a COVID-19 specific point, where doctors and patients consult online (3) SMS based messaging system to provide financial support to the community suffered amid COVID-19 lockdown through EHSAAS program [66].

The United Nations Development Programme (UNDP) Pakistan has partnered with Sehat Kahani, a health tech social enterprise with a network of 27 e-clinics across Pakistan, that connects patients to qualified doctors via a telemedicine based mobile and web solution— to accelerate the efforts to support Pakistan's healthcare system [67]. When Sehat Kahani app was initially launched the number of consultations were from 50 to 60 per day but now this figure has reached to 500–600. This app has more than 300 doctors who provide healthcare services free of cost to the patients [68]. In Pakistan during the Coronavirus outbreak many telemedicine mobile apps were introduced to battle with the adverse effects of COVID-19 scenarios. The famous telemedicine app known as Elaj Asan app is launched by AKHS and Aga Khan Development Network Digital Health Resource Centre (AKDN dHRC) in a joint venture. This app enables patients to interact with doctors and get appointment at one of three medical centers of AKUH. It facilitates patients to perform real-time, e-consultations and sharing of medical records with healthcare professionals in secure and private fashion. This app enables doctors to pre-screen their patients, examine through online face to face videos sessions, medicine prescription and maintaining patient's records. This telemedicine mobile health app has many other features like digital payment for healthcare services, convenient and easy access to healthcare services and providing instant feedback about their experiences [69]. Tele Polyclinic app is also reckoned as best choice of telemedicine app. This app was initially started by a young doctor belonging to Sindh. Its web version was launched in 2018 and app version was released during the COVID-19. During the period of lockdown, it played a good role in providing medical services to the patients. It is Android based application designed to connect doctors with patients to examine, test and treat them using digital infrastructure. Over 3000 doctors are registered and more than 300 specialists, who are providing medical care services without costing the patients via this app. This app is very useful for female patients to video call directly to lady doctor. This network has equal contribution from lady doctors and female patients always have the choice of taking help directly from lady doctors through phone or video calls whenever needed, Doctors have helped more than 50k patients through Tele Polyclinic [70].

Other popular mobile apps and digital platforms having telemedicine features are: MedIQ: Smart Healthcare, Corona Check, COVID CHECK PAKISTAN, COVID-19 Gov PK and AKUH Sehat Check and Marham.

### 4.6 Medical education and training apps

COVID-19 pandemic posed unprecedented challenges for the nursing and medical education [71]. During COVID-19, the mobile apps have been applied to provide medical education and training to the medical students, healthcare professionals, patients and citizens in Pakistan. Medical education and training apps allows the doctors to remain up to date about the medical world. According to the finding of this survey, we failed to find any specific medical training and online surgery related apps. This is due to availability of various videoconferencing apps like Zoom, Google Meet, and Microsoft Teams. Some of the mobile apps which are providing medical education are CoronaCheck and OpenWHO. CoronaCheck provide authentic and valuable information shared by experts to the users. These videos provide good information about the physical distancing, measures to adopt preventive measures, guidelines on wearing PPEs and detail of emergency contact numbers [72]. Similarly, OpenWHO app was launched by WHO interactive knowledge-transfer platform is providing online courses to expand the response to the health related emergencies. This paper helps the organization to deliver life-saving information to large numbers of stakeholders such as frontline responders, healthcare workers and decision makers [73].

### 4.7 Contact tracing apps

According to literature study, among the list of all mobile apps collected in this study, only one mobile app is found that designed to provide contact tracing feature and this app is known as COVID-19 Gov PK app. The less use of mobile based contact tracing is due to use to the use of militant-tracking technology for tracking and tracing patients. COVID-19 GOV PK mobile app provides contact tracing based on geo location. The most important feature of this app is “Radius Alert”. It helps in maintaining social distancing by keeping 1-m distance from the infected person. It has been available for use since April and has the record number of downloads in first week of its launching. Although, this app was criticized by a French hacker known as Elliot Alderson. According to him this app is not really providing contact tracing and it also detrimental to security and privacy issues like hardcoded passwords and insecure requests and downloading the exact coordinates of infected person while pressing radius alter button. Shabahat Ali Shah CEO of NITB in response to these remarks made it clear that coordinates and passwords are not the part of app workflow.

### 4.8 User satisfaction and feedback

**Fig. 5:**
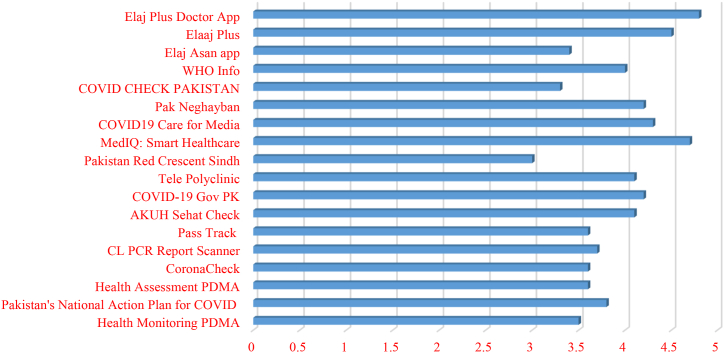
User feedback selected mobile apps.

In this research, we classified the mobile apps according to the feedback or responses received from the mobile users and subscribers. The user satisfaction of the mobile app can be judged by the user rating and comments provided in the comment section of the respective play stores. We collected the various responses and user rating of mobile apps from the play store to understand about the users’ perspective. The complete detail about the users rating is given in Fig. 5.

According to the data of Fig. 5, those COVID-19 mobile apps having user rating greater than 3.5 were considered as satisfactory by the mobile users. Some of apps such as Elaj Asan app, COVID Check Pakistan, Pakistan Red Crescent Sindh and Health Monitoring PDMA apps failed to satisfy the mobile users due to their low rating score values. While some of the mobile apps were highly appreciated by the mobile users. These mobile apps include Elaj Plus Doctor, MedIQ, Elaj Plus, Pak Neghayban, WHO Info and COVID-19 Gov PK.

### 4.9 Security, privacy and accessibility

**Fig. 6:**
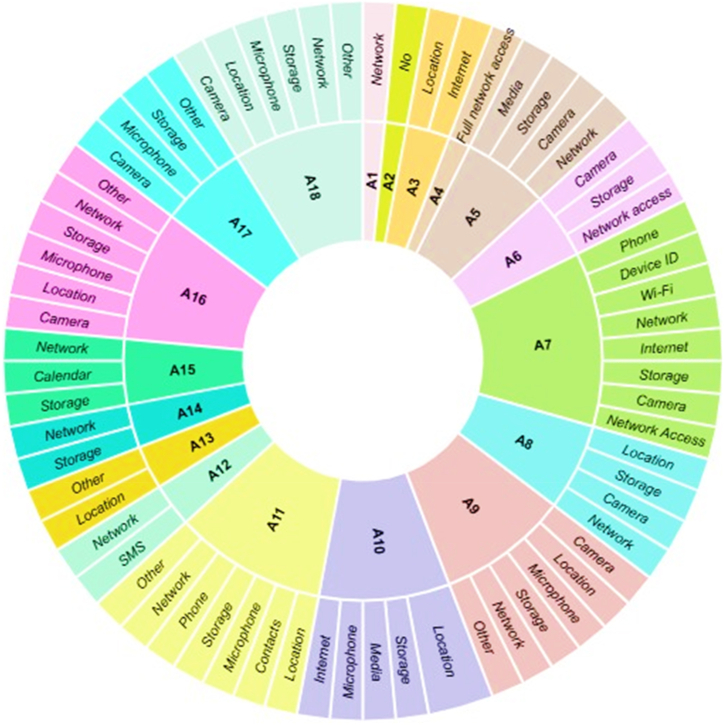
Access level of the selected mobile apps.

The healthcare system is on the top of the list of targeted systems by the cybercriminals around the world [74]. The nature of healthcare data is complex due to its storage in multi-table database in comparison to other traditional data [75]. Therefore, the security of COVID-19 mobile apps using the healthcare along with state-of-the-art fiber optic technology is becomes major concern [76]. During the pandemic, it has also been observed that COVID-19 GOV PK mobile app is vulnerable to the security risks. This app get access to the geographic locations of the mobile user or patient by downloading the exact co-ordinates during the process of contact tracing or radius alert feature. Similarly, the contact tracing process COVID-19 Gov Pk is using centralized approach. Pass Track app, MedIQ: Smart Healthcare, and AKUH Sehat Check mobile apps have more secure features in comparison to the other selected apps in this study. These apps provide security in the sense that data collected by these mobile apps are never shared with the third parties. These apps provide a secure way of transferring data and they give a full control to the users to remove data collected by these mobile apps. MedIQ: Smart Healthcare is also collecting data regarding personal information, device ID, photos, videos etc. The level of accessibility of each selected mobile apps is given in Fig. 6.

## 5 Meta-data analysis of selected COVID-19 apps

**Fig. 7:**
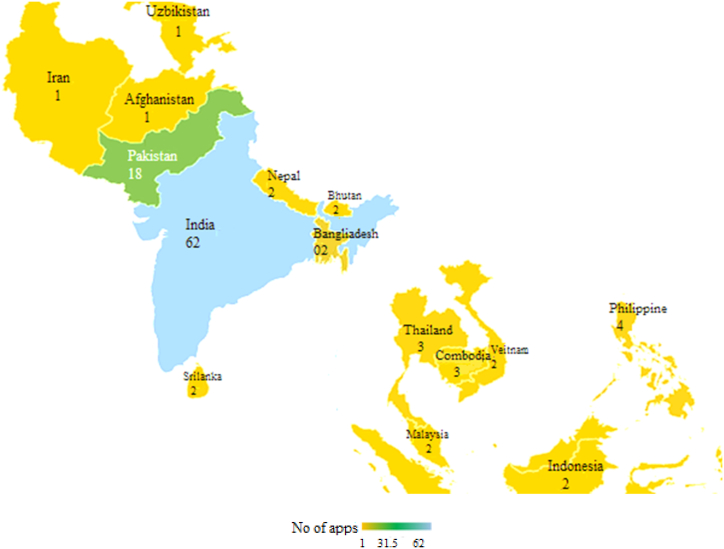
Comparing the number of COVID-19 apps of Pakistan with South Asian countries.

After the application of eligibility/inclusion criteria, we selected 18 mobile apps that were solely intended to contain the COVID-19 pandemic as previously discussed. These mobile apps were investigated for the different features and functions. According to our analysis, it has been observed that mobile apps with self-assessment features are (n = 6, 33 %), home monitoring featured apps (n = 9, 50 %), medical education apps (n = 1, 6 %), COVID awareness apps (n = 9, 50 %), telemedicine apps (n = 9, 50 %), Coronavirus surveillance apps (n = 7, 39 %) and contact tracing apps (n = 1, 6 %). The complete detail of feature mapping with mobile apps is given in Table 6. In this table, the selected mobile apps are written against the features of the assessment criteria. The frequency indicates the number of features and the percentage shows the feature percentage of every mobile app concerning the assessment criteria. A comparative analysis is performed to know about the number of mobile apps released by different countries during COVID-19. Among the list of South Asian countries, Pakistan has the second highest number of apps after India as given in Fig. 7.

**Table 6:**
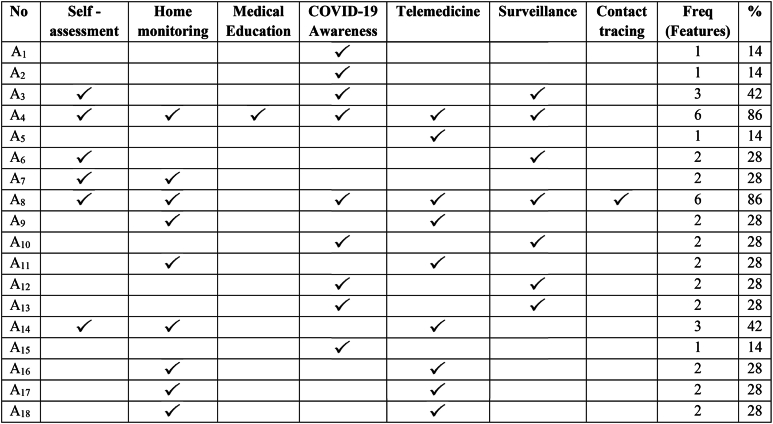
Mobile apps feature mapping.

**Fig. 8:**
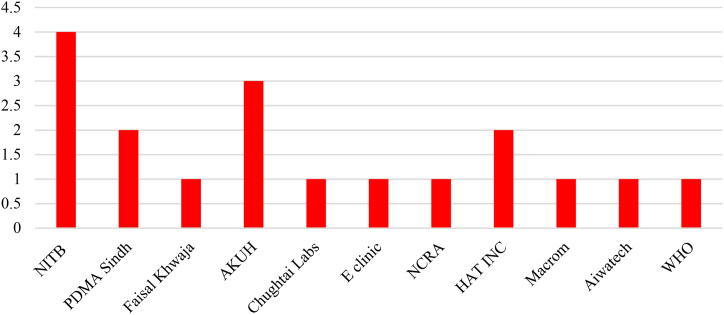
Major mobile app developers.

Both government and private sectors collectively contributed towards developing COVID-19 apps but the role of government cannot be sidelined. NITB is the leading app developer among the contestants. The complete detail is given in Fig. 8.

**Fig. 9:**
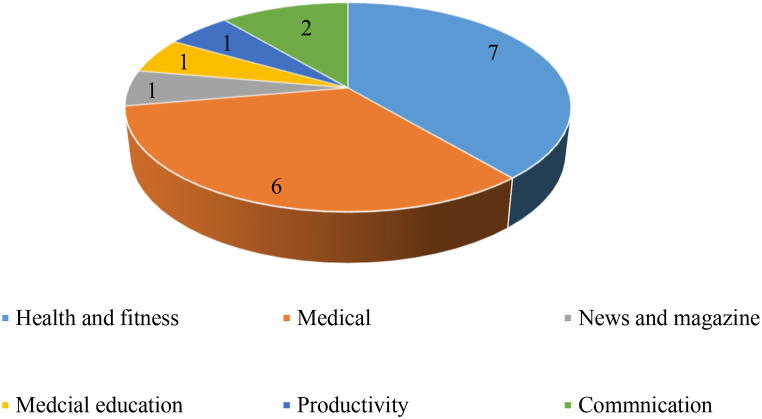
Different categories of selected mobile apps.

Finally, selected mobile apps were analyzed and checked for the type of services and features provided by them. In this list of selected apps, 07 apps were focusing on health and fitness by 06 medical apps. The complete categories of mobile apps are given in Fig. 9.

**Fig. 10:**
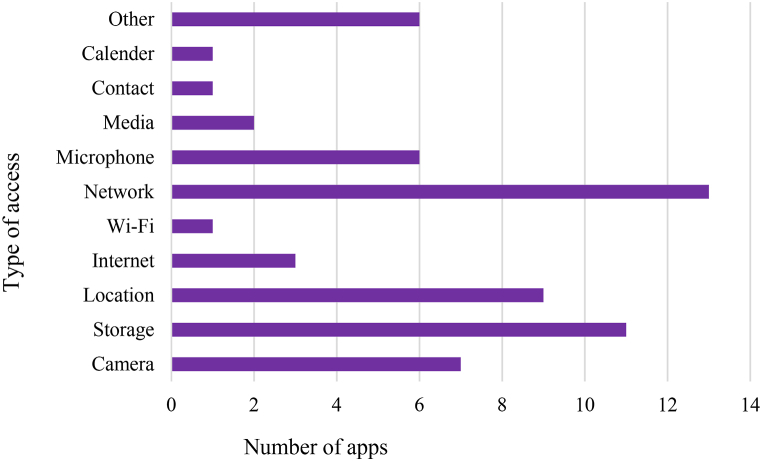
Permission level of mobile apps.

**Fig. 11:**
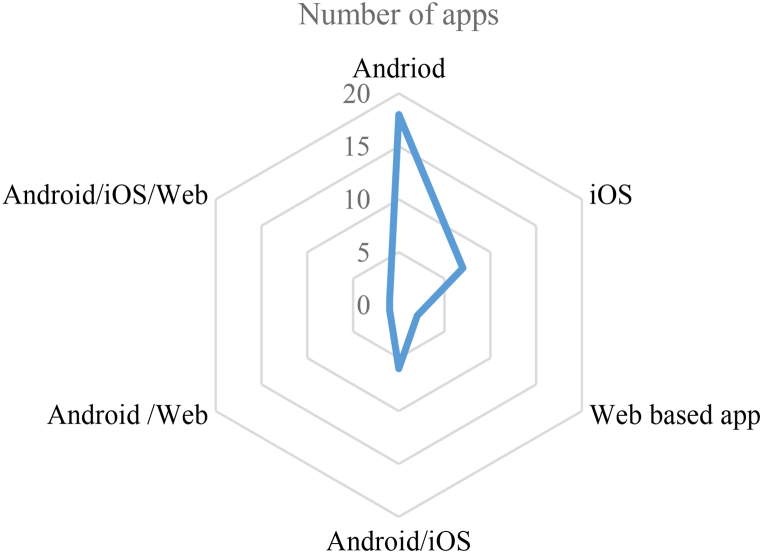
Platform-wise apps detail.

Similarly, we also analyzed mobile apps for the type of access or permission level to the mobile phone. The majority of mobile apps ask for access to the network, location, and storage. The complete picture of access level by the number of apps in our selected apps is given in Fig. 10. The detail of different platforms supported by the selected mobile health apps is given in Fig. 10. The majority of apps use using Android platform for running as shown in Fig. 11.

**Fig. 12:**
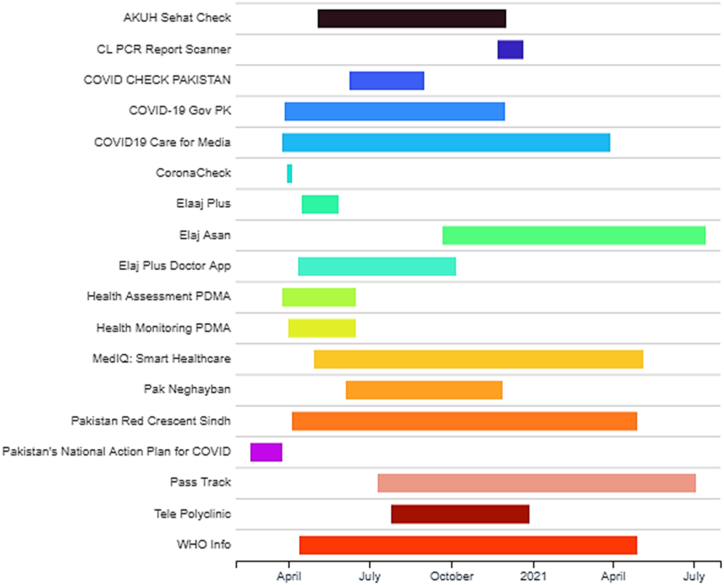
Gantt chart of release and update dates of mobile apps during COVID-19 in Pakistan.

It is also important to investigate the date of release and update of every mobile app. For this purpose, we created a Gantt chart to visualize the release and updating dates and to build a timeline for every mobile app. The complete timeline of releasing and updating dates of COVID-19 apps in Pakistan is given in Fig. 12.

## 6 Discussion

This survey is mainly intended to provide a comprehensive and in-depth analysis of various mobile health apps introduced during the COVID-19 pandemic in Pakistan. This study evaluates and discusses the different apps based on the criteria features and we believe it is helpful for the various stakeholders such as mobile app subscribers, app developers, and healthcare professionals. The mobile subscribers will find the most desired apps according to their feature requirements as it covers every mobile app and synthesizes the comments/reviews written by reviewers or users. This study will also assist mobile app designers or developers in understanding the existing features and updating mobile apps according to COVID-19-related features. It is also helpful for them to understand the current position of their apps concerning the COVID-19-specific features. Similarly, this study will be fruitful for healthcare personnel working in the hospital to pick the right choice of mobile apps for healthcare tasks and services.

Some of the results drawn by this survey, also show that most mobile apps have claimed the features in the Play stores but in reality, these features do not exist or are partially available. For example, the COVID-19 Gov PK app has a written description of the contact tracing feature, but this feature is not fully functionally available. But, still, according to the findings of this study, the COVID-19 Gov PK mobile app is reckoned as the most ideal choice of mobile application to deal with the effects of the pandemic. This app provides more features related to the COVID and hence, it is recommended for all stakeholders. We hope the results of this study might be very effective for the stakeholders to improve their apps during the pandemic. The results of this study are also helpful for the governmental institutions that are working as monitoring and inspecting units during the pandemic. It will help them to understand the significance of mobile technology and to collaborate with developers to enhance the existing mobile technology to overcome the effects of a pandemic.

According to the results of this survey, it has also been learned that many healthcare-related apps that were previously available in the app stores, were also upgraded to bring the most updated features related to the pandemic. Major software development companies rather than introducing new mobile apps focused on upgrading or updating their existing mobile apps. COVID-19 Awareness, medical education, and surveillance are the most common features that were added to the many mobile apps. However, according to the results of this study, we found that none of the mobile apps has been found that was bringing the most vital feature of contact tracing. The major reason for not bringing this kind of feature due to budgetary issues and some other available technology applications. Although, the” radius alert” feature of the COVID-19 Gov Pk app, is important for contact tracing but its usage was significantly affected by privacy and security issues.

## 7 Limitations

There are some limitations of this review article such as it is likely that some of the mobile applications may not be retrieved by the defined keywords that have been applied for searching the mobile apps in play stores. The keywords include only COVID or Corona related terms so chance of skipping apps is likely due to the different nomenclatures of apps. The search procedure was conducted from June 09, 2021 to June 30, 2021, therefore it is likely that some more features might be added, or some new apps might have been released after the end date.

There are some mobile apps that were released before COVID-19 pandemic, but they are updated to include the COVID-19 related features such apps are not included in our study. The features selected in this study for mobile applications are not necessary to be considered as benchmark as there are many features that may be important to other authors or app developers. Mobile apps are only studied for COVID-19 related features, it is likely that some mobile apps might be providing better features and performance but it may not be able to meet other most essential features like information provision [77], security and privacy [27], design and visual appearance [77,78] and usability.

## 8 Conclusion

The major success of Pakistan related to handling the COVID-19 outbreak is because of the transformation and adoption of digital technologies. Among these technologies, the role of smartphone mobile applications is of paramount importance. During COVID-19, both government and private sectors in Pakistan developed an array of mobile apps to tackle the impacts of COVID-19. Many applications have been proposed with a variety of COVID-19-related features. It is indispensable to highlight the features and functions of mobile apps so that mobile subscribers may find it easy to get the best app according to their needs and expectations. This research work attempts to highlight all the functions and features of mobile apps targeted toward the COVID-19 pandemic in Pakistan. For this purpose, we set two criteria eligibility criteria and assessment criteria. The purpose of the former criteria is to filter the mobile apps collected from three sources- Google Play Store, Apple App Store, and web sources. The objective of inclusion or eligibility criteria is to get the most relevant apps that are specially designed for the COVID-19 pandemic. The mobile applications selected for assessment are based on the second criterion known as the assessment criterion. The major indicators/features of this criterion are awareness, home monitoring, medical education, self-assessment, telemedicine, and contact tracing. All mobile apps are evaluated and discussed in light of the features defined by this criterion. A complete and in-depth analysis has been performed to know about the current research trend, understand the features and functions of every mobile app and the role of each app towards mitigation of the COVID-19 impacts in Pakistan. This study is helpful by providing a guideline for readers and researchers to know about the current research trends and to provide future research directions for mobile-based technologies attempted towards COVID-19 in Pakistan.

As, this study compares mobile apps based on theoretical ground. In future work, we are looking forward to build an empirical intelligent evaluation framework by designing more sophisticated evaluation criteria. We are also looking forward to consider more features such as security, privacy, usability etc. in the proposed criteria.

## Data availability

All data generated or analyzed during this study are included and displayed in this article.

## CRediT authorship contribution statement

**Habib Ullah Khan:** Supervision, Resources, Project administration, Funding acquisition, Data curation, Conceptualization. **Yasir Ali:** Writing – review & editing, Writing – original draft, Visualization, Validation, Methodology, Investigation, Formal analysis, Data curation. **Muhammad Azeem Akbar:** Funding acquisition, Resources. **Faheem Khan:** Funding acquisition, Resources, Software, Writing – review & editing.

## Declaration of competing interest

The authors declare that they have no known competing financial interests or personal relationships that could have appeared to influence the work reported in this paper.
